# Supplementary material for: Low carbon renewable natural gas production from coalbeds and implications for carbon capture and storage
Source: Nat Commun. 2017 Sep 18;8:568. doi: 10.1038/s41467-017-00611-7 (PMC5603537; doi:10.1038/s41467-017-00611-7)
Supplement: Supplementary file 1 — Supplementary Information [file 41467_2017_611_MOESM1_ESM.pdf]

Description of Supplementary Files

File Name: Supplementary Information
Description: Supplementary Figures and Supplementary Tables

File Name: Peer Review File

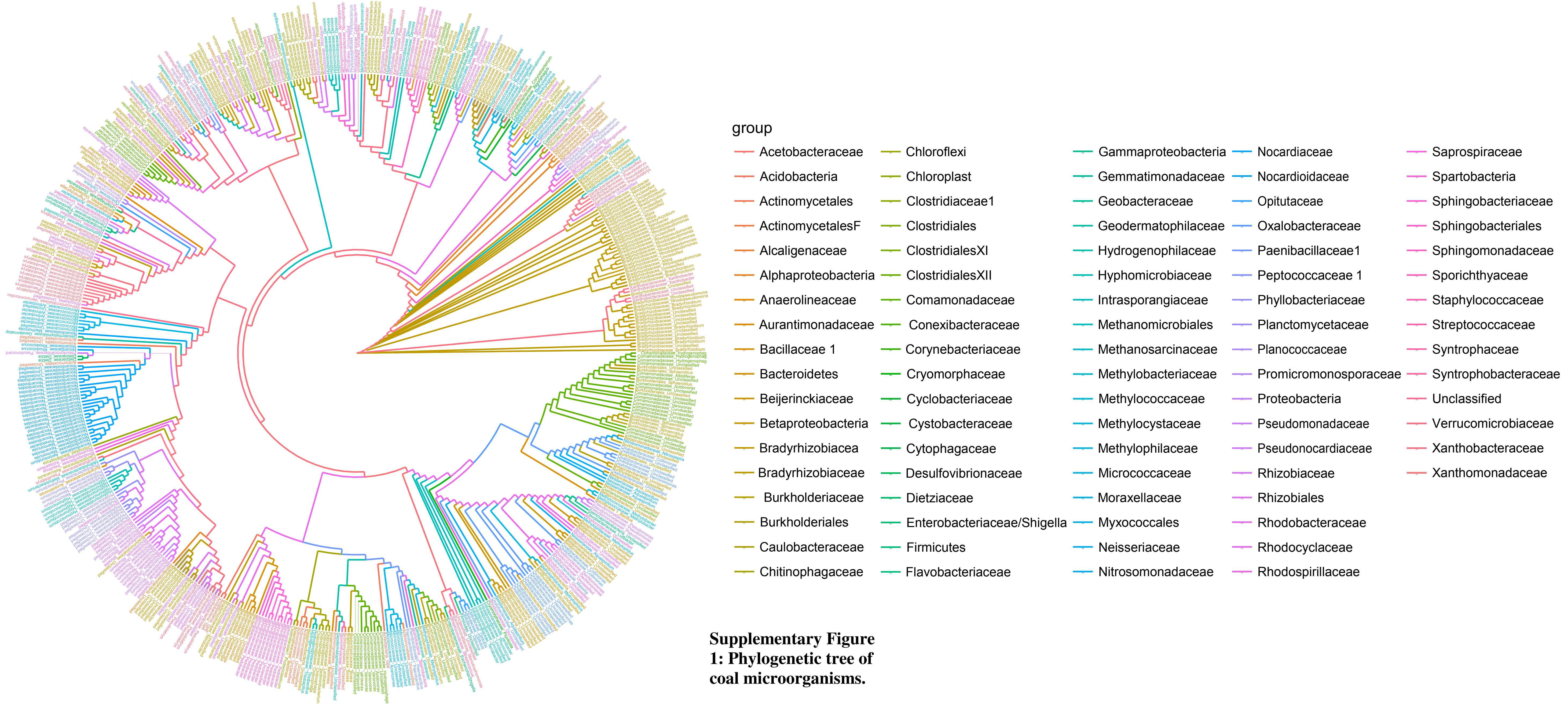

Supplementary Figure 1: Phylogenetic tree of coal microorganisms.

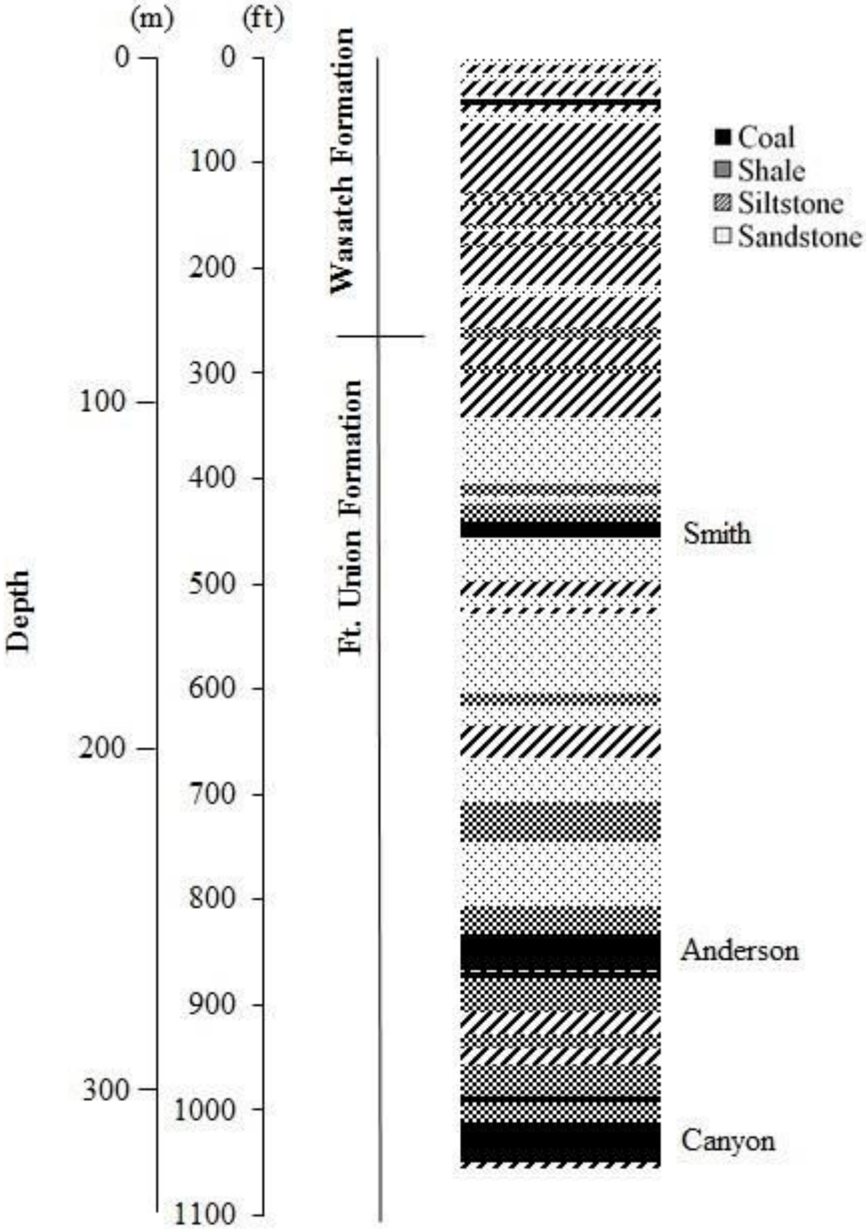

Supplementary Figure 2: Stratigraphic column interpreted from petrophysical data collected from well Fed 41-18. The stratigraphic column broadly classifies the geologic layers coal, shale, sandstone, and siltstone starting at a depth of 18 feet to 1053 feet (5~321 meters) below ground surface (bgs). The sampled well has a total depth of 1,053 feet (321 meters) bgs and intercepted three major coal seams: Smith, Anderson, and Canyon coals. The coal sample was collected from Canyon coal in the Wyodak coal zone with depth between 312-321 meters bgs.

Supplementary Table 1: Conversion efficiencies for plant-derived carbohydrates

Treatment	Gal	Man	Glu	Cel	Xyl	Ara	Neg. Ctrl	AA Ctrl	H_2/CO_2 Ctrl
Concentration (g L^{-1})	1.8	1.8	1.8	3.4	1.5	1.5	0	0.52	1
Actual CH_4 (ml)	13.5	14.6	12.7	1.2	13.3	13.7	0	3.7	0.1
Theoretical CH_4 (ml)	29.3	30.6	29.5	56	25	24	0	10	10
Theoretical specific yield (mol mol^{-1} substrate)	2.4	2.4	2.4	5.8	2	2	0	0.8	1
Conversion efficiency (%)	46 ± 6.1	55 ± 6.9	42 ± 2.6	2 ± 2.1	54 ± 0.9	55 ± 1.5	0 ± 0	50 ± 10.7	1 ± 0.2
Stat. similarity	a	a	a	b	a	a	b	a	b
Day of peak	77	65	65	106	83	73	0	83	83

Supplementary Table 2: Coal analyses. Ash (ASTM D-5142), Sulfur (ASTM D-4239), and energy content (ASTM D-5865) of coals

	6-Carbon	Control incubated	5-Carbon	Control non-incubated
Ash, wt%	9.27	9.95	11.785	10.905
Sulfur, wt%	0.298	0.305	0.283	0.298
Energy Content, Btu lb^{-1}	11,857	11,682	11,463	11,440

Supplementary Table 3: Ultimate analysis and heating value of the sampled coal among the Canyon coal seam of the Wyodak coal zone in the PRB

Ultimate Analysis of coal	Moisture-free basis	Proximate Analysis of coal	Moisture-free basis
Carbon	65.1%	Volatile Matter	37.1%
Hydrogen	4.4%	Fixed Carbon	48.4%
Oxygen	14.9%	Ash	14.5%
Nitrogen	0.9%	Total	100.0%
Sulfur	0.2%	Btu lb^{-1}	11,440
Ash	14.5%		
Total	100.0%		

Supplementary Table 4: Coal microcosm treatments and controls

Substrate	Concentration	Chemical Formula/M.W.
D-galactose	10 mM	$C_6H_{12}O_6$/180.16
D-mannose	10 mM	$C_6H_{12}O_6$/180.16
D-glucose	10 mM	$C_6H_{12}O_6$/180.16
D-cellobiose	10 mM	$C_{12}H_{22}O_{11}$/342.3
D-xylose	10 mM	$C_5H_{10}O_5$/150.13
L-arabinose	10 mM	$C_5H_{10}O_5$/150.13
Mix:galactose, mannose, glucose, cellobiose, xylose, arabinose	10 mM	mix: equimolar of (galactose, mannose, glucose, cellobiose, xylose, and arabinose)
Negative control: (no substrate)	-	-
Positive control: (acetic acid)	10 mM	$C_2H_4O_2$/60.02
Positive control: Ultra High Purity 99.999% H_2 gas and $NaHCO_3$	12 mM (HCO_3^-)	-
